# Emerging Role of Plant-Based Dietary Components in Post-Translational Modifications Associated with Colorectal Cancer

**DOI:** 10.3390/life13020264

**Published:** 2023-01-18

**Authors:** Carmen Rodríguez-García, Francisco Gutiérrez-Santiago

**Affiliations:** 1 Department of Health Sciences, Faculty of Experimental Sciences, University of Jaen, Campus las Lagunillas s/n, 23071 Jaen, Spain; 2 University Institute of Research in Olive Groves and Olive Oils, University of Jaen, Campus las Lagunillas s/n, 23071 Jaen, Spain; 3 Department of Experimental Biology, Faculty of Experimental Sciences, University of Jaen, Campus las Lagunillas s/n, 23071 Jaen, Spain

**Keywords:** cancer risk factor, flavonoids, lignans, phenols, phosphorylation, prognosis, natural products

## Abstract

Colorectal cancer (CRC) is one of the most common cancers worldwide. Its main modifiable risk factors are diet, alcohol consumption, and smoking. Thus, the right approach through lifestyle changes may lead to its prevention. In fact, some natural dietary components have exhibited chemopreventive activity through modulation of cellular processes involved in CRC development. Although cancer is a multi-factorial process, the study of post-translational modifications (PTMs) of proteins associated with CRC has recently gained interest, as inappropriate modification is closely related to the activation of cell signalling pathways involved in carcinogenesis. Therefore, this review aimed to collect the main PTMs associated with CRC, analyse the relationship between different proteins that are susceptible to inappropriate PTMs, and review the available scientific literature on the role of plant-based dietary compounds in modulating CRC-associated PTMs. In summary, this review suggested that some plant-based dietary components such as phenols, flavonoids, lignans, terpenoids, and alkaloids may be able to correct the inappropriate PTMs associated with CRC and promote apoptosis in tumour cells.

## 1. Introduction

Colorectal cancer (CRC) is currently the second type of cancer with the highest mortality rate in the population according to Global Cancer Statistics 2020 [1]. Metastatic CRC has a poor prognosis, with less than a 15% of five-year survival rate [2]. Its carcinogenesis is a process of many years of development and some early life risk factors are important contributors [3]. Among them, cigarette smoking, obesity, and a sedentary lifestyle are closely related to CRC incidence [4,5]. However, its quickly increasing incidence is mainly due to lifestyle westernization associated with changes in dietary behaviour such as heavy alcohol consumption and diets rich in sugars, saturated fats, and red and processed meat [6]. Thus, some protective lifestyle factors against CRC include a diet rich in minerals and vitamins, dairy, dietary fibre, fish, vegetables, and fruits. An alternative strategy for CRC prevention is the use of a chemopreventive supplement providing greater individual exposure to some nutrients than can be obtained from the diet (such as phytochemicals) [7].

The pathogenesis of CRC is a complex multi-stage process which includes gut microbiota imbalances, cell DNA disruption, and carcinogenic signalling pathways activation [8]. The aetiology underlying the mechanism of action of specific nutrients in CRC has been mainly attributed to their anti-inflammatory and antioxidant properties, and their modulation of gut microbiota populations, maintaining gut homeostasis and regulating the host immune response [9,10]. However, their effects on epigenetic modulation associated with CRC pathogenesis remains unknown. There is increasing evidence that the disruption of epigenetic control over gene expression has an important role in carcinogenesis [11,12,13,14]. Together with non-coding RNAs and DNA methylation, histone and protein post-translational modifications (PTMs) have an important role in carcinogenesis and gene regulation [15,16]. PTMs occur once the mRNA has been translated into the protein sequence in the ribosomes and produce marginal chemical modifications to lipoproteins and native proteins. Among these modifications, PTMs may mark proteins for degradation, inhibit or promote interactions with other proteins, redirect cellular protein localization, and modify enzyme activity [17,18]. Most PTMs are reversible, so normal cells use them as a switch to control proliferating or quiescent cells [19]. The role of PTMs in the onset and progression of diseases such as cancer has been investigated. Their involvement in the process of carcinogenesis could be due to their function in processes such as the cell cycle, cell survival, and cell proliferation [20]. Therefore, PTM-focused analysis of enzyme phosphorylation and the involvement of protein kinases in cancer formation and progression have led to the use of PTM-based therapeutic approaches (i.e., tyrosine kinase inhibitors) [21,22]. Furthermore, in the case of CRC, PTMs develop key role-playing as a tight junction protein and regulate the epithelial barrier function [23,24]. Thus, PTMs may be essential to work with the external impact and could provide an excellent opportunity for intervention through feeding and promoting clinical strategies for CRC patients regarding predictive, preventive, and personalized medicine. Overall, the aims of this article were (1) to summarize the main PTMs involved in CRC development; (2) to identify the connection between the main PTMs involved in CRC via the Search Tool for the Retrieval of Interacting Proteins 11 (STRING 11) [25]; and (3) to review the plant-based dietary components that can modulate these modifications.

## 2. Post-Translational Modifications in Colorectal Cancer

**Figure 1:**
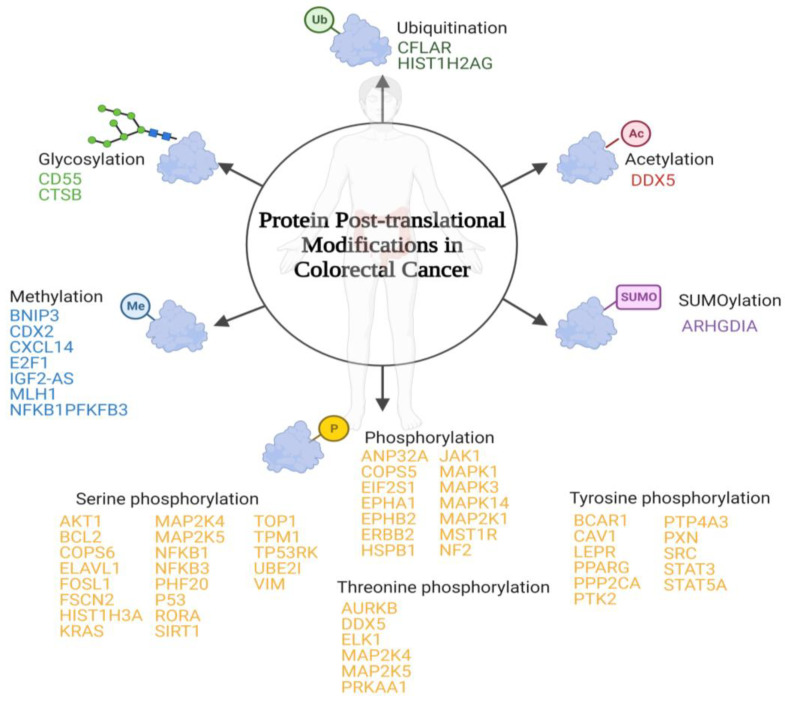
Schematic representation of the main post-translational modifications in colorectal cancer. Below each post-translational modification is a list of the identified proteins that suffer inappropriate post-translational modifications associated with colorectal cancer.

PTMs are protein-specific modifications that control many physiological processes to ensure the dynamic and quick response of cells to intracellular and extracellular stimuli [26]. Any proteome protein may be modified post-translationally or during translation. These reversible modifications may alter not only the protein’s stability, conformation, and charge state, but also its function modulating its intracellular conformation, its interactions, and the life span of the target protein [27]. In some cases, PTMs are inadequate and modulate positively some signal transduction pathways that are involved in tumourigenesis regulation and cancer development [28]. To date, more than 450 unique protein modifications have been described, including ubiquitination, acylation, SUMOylation, methylation, and phosphorylation [29]. In the case of CRC, the most important modifications involved have been summarized below (Figure 1).

### 2.1. SUMOylation

Small ubiquitin-like modifiers (SUMO) are covalently attached to lysine residues [30]. The downregulated SUMOylation in lysine 138 of Rho GDP-dissociation inhibitor 1 has been observed in CRC cell lines (Table 1). This protein is involved in Rho GTPases signalling regulation [31].

### 2.2. Glycosylation

A carbohydrate is attached to specific proteins. In mammals, there are two types: (1) O-glycosylation, where glycosyl groups are connected to tyrosine, hydroxylysine, serine, or threonine side chains with glycosidic linkages by glycosyltransferases, and (2) *N*-glycosylation, where glycosyl groups are connected to Asn side chains with amide linkages by oligosaccharyltransferase [32,33]. The upregulation of this PTM in complement decay-accelerating factor and cathepsin B has been identified in tumour tissue samples of CRC patients (Table 1) [34,35].

### 2.3. O-GlcNAcylation

There is a covalent attachment of *N*-acetylglucosamine residue O-linked to the hydroxyl group of threonine and serine residues of multiple cytosolic and nuclear proteins [36,37]. The upregulation of O-GlcNAcylation in ATP-dependent RNA helicase DDX5 has been associated with CRC in cell lines and murine models (Table 1) [38].

### 2.4. Ubiquitination

There is an attachment of ubiquitin molecules to the lysine residue of the substrate proteins. This process is based on an enzymatic cascade of ubiquitin-activating, ubiquitin-conjugating, and ubiquitin-ligase enzymes [39,40]. There have been two ubiquitination-susceptible proteins identified that are related to CRC (Table 1): (1) caspase homolog that is an apoptosis regulator [41] and (2) histone H2A type 1 that is involved in chromosomal stability, DNA replication, and DNA repair [42].

**Table 1:** Post-translational glycosylation, O-GlcNAcylation, SUMOylation, and ubiquitination associated with CRC.

Protein Name	Gene Name	PTM	PTM Site	Type	Ref
Rho GDP-dissociation inhibitor 1	ARHGDIA	SUMOylation	K138	Downregulated	[31]
Complement decay-accelerating factor	CD55	O-linked glycosylation	NA	Upregulated	[34]
Cathepsin B	CTSB	Glycosylation	NA	Upregulated	[35]
Probable ATP-dependent RNA helicase DDX5	DDX5	O-GlcNAcylation	NA	Upregulated	[38]
Caspase homolog	CFLAR	Ubiquitination	K195	Upregulated	[41]
Histone H2A type 1	HIST1H2AG	Ubiquitination	NA	Upregulated	[42]

NA: data not available; K: lysine.

### 2.5. Methylation

Methylation occurs mainly in arginine or lysine residues. One of the most biologically important roles of methylation is in histone modification [43]. Among the different proteins that suffer dysregulated post-translational methylation associated with CRC (Table 2), the one that is involved in cell growth suppression has downregulated methylation (putative insulin-like growth factor 2 antisense gene protein) [44,45]. The other proteins identified have an upregulated methylation, among them are (1) BCL2/adenovirus E1B 19 kDa protein-interacting protein 3 that is involved in apoptosis [46]; (2) homeobox protein CDX-2 that is involved in the transcriptional regulation of different genes expressed in the intestine [47]; (3) C-X-C motif chemokine 14 that is involved in immunoregulatory and inflammatory processes [48]; (4) transcription factor E2F1 that participates in the cell cycle [49]; (5) DNA mismatch repair protein Mlh1 that participates in DNA repair [50]; (6) nuclear factor NF-kappa-B p105 subunit that is a pleiotropic transcription factor involved in several signal transduction events which are initiated by stimuli such as oxidative stress or inflammation [51]; and (7) 6-phosphofructo-2-kinase/fructose-2,6-bisphosphatase 3 that is an essential protein for cell cycle progression and apoptosis prevention [52].

**Table 2:** Post-translational methylation associated with CRC.

Protein Name	Gene Name	PTM Site	Type	Ref
BCL2/adenovirus E1B 19 kDa protein-interacting protein 3	BNIP3	NA	Upregulated	[53]
Homeobox protein CDX-2	CDX2	NA	Upregulated	[54]
C-X-C motif chemokine 14	CXCL14	T72	Upregulated	[55]
Transcription factor E2F1	E2F1	K109/111/113	Upregulation	[56]
Putative insulin-like growth factor 2 antisense gene protein	IGF2-AS	NA	Downregulated	[45]
DNA mismatch repair protein Mlh1	MLH1	NA	Upregulated	[57]
Nuclear factor NF-kappa-B p105 subunit	NFKB1	K218/221	Upregulated	[58]
6-phosphofructo-2-kinase/fructose-2,6-bisphosphatase 3	PFKFB3	R131/134	Upregulated	[59]

NA: data not available; K: lysine; R: arginine.

### 2.6. Phosphorylation

Phosphorylation is the most prevalent and widely studied type of PTM. It is inversely regulated by phosphatases and protein kinases in the amino acids’ hydroxyl tyrosine, threonine, or serine [32,60]. In the case of CRC, inadequate PTMs have been identified in the following proteins (Table 3): (1) acidic leucine-rich nuclear phosphoprotein 32 family member A that is involved in cell growth [61]; (2) COP9 signalosome complex subunit 5 that develops an important role in the degradation of cyclin-dependent kinase inhibitor [62]; (3) eukaryotic translation initiation factor 2 subunit 1 that is a translation initiation factor [63]; (4) ephrin type-A receptor 1 and ephrin type-B receptor 2 that are members of the ephrin receptor subfamily of the protein tyrosine kinase family [64]; (5) receptor tyrosine-protein kinase erbB-2 that is a member of the epidermal growth factor receptor family [65]; (6) heat shock protein beta-1 which plays an important role in cancer cells proliferation [66]; (7) tyrosine-protein kinase JAK1 that is a tyrosine kinase of the non-receptor type [67]; (8) mitogen-activated protein kinase 1, 3, and 14 that are serine/threonine kinases that are essential components of the MAP kinase signal transduction pathway [68,69,70,71]; (9) dual specificity mitogen-activated protein kinase kinase 1 which acts as an essential component of the MAP kinase signal transduction pathway [72]; (10) macrophage-stimulating protein receptor that is a tyrosine kinase receptor [73]; and (11) merlin that plays a pivotal role in tumour suppression through apoptosis promotion [74].

Table 4 summarizes the inadequate post-translational serine, threonine, and tyrosine phosphorylation in proteins that have been associated with CRC. With regards to phosphorylation, the main affected proteins and their functions are the following (Table 4).

### 2.7. Serine Phosphorylation

Serine phosphorylation includes proto-oncogene c-Ak and Fos-related antigen 1 that regulates many processes including proliferation cell survival, growth, and angiogenesis [75,76]; apoptosis regulator Bcl-2 that is a regulator of apoptosis [77]; COP9 signalosome complex subunit 6 which is a component of the COP9 signalosome complex [78]; ELAV-like protein 1 that stabilizes mRNAs and regulates gene expression [79]; fascin-2 that acts as an actin bundling protein [80]; histone H3.1 which plays a central role in transcription regulation and DNA repair [81]; Kirsten rat sarcoma virus which is involved in the propagation of growth factors [82]; MAP kinase kinase 4 and 5 that are dual specificity protein kinase which act as an essential component of the MAP kinase signal transduction pathway [83,84]; NFKB1 and NFKB3 which are pleiotropic transcription factors involved in several signal transduction [85,86]; PHD finger protein 20 that contributes to p53 stabilization after DNA damage [87]; cellular tumour antigen p53 that acts as a tumour suppressor [88]; nuclear receptor ROR-alpha which is a key regulator of glucose metabolism [89]; sirtuin 1 that is an intracellular regulatory protein [90]; DNA topoisomerase 1 that releases the supercoiling tension of DNA introduced during the DNA replication [91]; tropomyosin-1 which is a member of the tropomyosin family of highly conserved proteins [92]; TP53-regulating kinase which is a protein kinase that phosphorylates ‘Ser-15’ of p53/TP53 protein [93]; SUMO-protein ligase that is essential for nuclear architecture and chromosome segregation [94]; and vimentin which is responsible for maintaining cell shape and stabilizing cytoskeletal interactions [95].

**Table 4:** Post-translational serine, threonine, and tyrosine phosphorylation associated with CRC.

Protein	Gene Name	PTM Site	Type	Ref
Serine Phosphorylation				
Proto-oncogene c-Akt	AKT1	S473	Upregulated	[75]
Apoptosis regulator Bcl-2	BCL2	S87	Upregulated	[77]
COP9 signalosome complex subunit 6	COPS6	S148	Upregulated	[78]
ELAV-like protein 1	ELAVL1	S318	Upregulated	[79]
Fos-related antigen 1	FOSL1	S252; S265	Upregulated	[76]
Fascin-2	FSCN2	S39	Upregulated	[80]
Histone H3.1	HIST1H3A	S28	Upregulated	[81]
Kirsten rat sarcoma virus	KRAS	S181	Upregulated	[82]
MAP kinase kinase 4	MAP2K4	S257	Upregulated	[83]
MAP kinase kinase 5	MAP2K5	S311	Upregulated	[84]
Nuclear factor NF-kappa-B p105 subunit	NFKB1	S536	Upregulated	[85]
Nuclear factor NF-kappa-B p65 subunit	NFKB3	S276	Upregulated	[86]
PHD finger protein 20	PHF20	S291	Upregulated	[87]
Cellular tumour antigen p53	P53	S15	Upregulated	[88]
Nuclear receptor ROR-alpha	RORA	S35	Downregulated	[89]
Sirtuin 1	SIRT1	S27	Upregulated	[90]
DNA topoisomerase 1	TOP1	S506	Upregulated	[91]
Tropomyosin-1	TPM1	S283	Upregulated	[92]
TP53-regulating kinase	TP53RK	S250	Upregulated	[93]
SUMO-protein ligase	UBE2I	S71	Upregulated	[94]
Vimentin	VIM	S72	Upregulated	[95]
Threonine Phosphorylation				
Aurora kinase B	AURKB	T232	Upregulated	[96]
Probable ATP-dependent RNA helicase DDX5	DDX5	T564/446	Upregulated	[97]
ETS domain-containing protein Elk-1	ELK1	T417	Upregulated	[98]
Dual specificity mitogen-activated protein kinase kinase 4	MAP2K4	T261	Upregulated	[83]
MAP kinase kinase 5	MAP2K5	T315	Upregulated	[84]
5’-AMP-activated protein kinase catalytic subunit alpha-1	PRKAA1	T183	Downregulated	[99]
Tyrosine Phosphorylation				
Breast cancer anti-estrogen resistance protein 1	BCAR1	Y12; Y128	Upregulated	[100,101]
Caveolin-1	CAV1	Y14	Upregulated	[102]
Leptin receptor	LEPR	Y1141	Upregulated	[103]
Peroxisome proliferator-activated receptor gamma	PPARG	Y102	Upregulated	[104]
Serine/threonine-protein phosphatase 2A catalytic subunit alpha isoform	PPP2CA	Y307	Upregulated	[105]
Focal adhesion kinase 1	PTK2	Y397; Y407; Y925	Downregulated	[106,107]
Protein tyrosine phosphatase type IVA 3	PTP4A3	Y53	Upregulated	[108]
Paxillin	PXN	Y88	Upregulated	[109]
Proto-oncogene tyrosine-protein kinase Src	SRC	Y419	Upregulated	[110]
Signal transducer and activator of transcription 3	STAT3	Y705	Upregulated	[111,112]
Signal transducer and activator of transcription 5A	STAT5A	Y694	Downregulated	[113]

S: serine; T: threonine; Y: tyrosine.

### 2.8. Threonine Phosphorylation

Threonine phosphorylation includes Aurora kinase B which is a serine/threonine-protein kinase component of the chromosomal passenger complex [96]; probable ATP-dependent RNA helicase DDX5 which is involved in the alternative regulation of pre-mRNA splicing [97]; ETS domain-containing protein Elk-1 which is a transcription factor that binds to purine-rich DNA sequences [98]; dual specificity mitogen-activated protein kinase kinase 4 which is an essential component of the MAP kinase signal transduction pathway [83]; MAP kinase kinase 5 that acts as a scaffold for the formation of a ternary MAP3K2/MAP3K3-MAP3K5-MAPK7 signalling complex [84]; and 5′-AMP-activated protein kinase catalytic subunit alpha-1 which is the catalytic subunit of AMP-activated protein kinase that plays a key role in regulating cellular energy metabolism [99].

### 2.9. Tyrosine Phosphorylation

Tyrosine phosphorylation includes breast cancer anti-estrogen resistance protein 1 which plays a central role in cell adhesion [100,101]; caveolin-1 that act as a scaffolding protein within caveolar membranes [102]; leptin receptor that mediates leptin central and peripheral effects [103]; peroxisome proliferator-activated receptor gamma that is a nuclear receptor [104]; serine/threonine-protein phosphatase 2A catalytic subunit alpha isoform which is the major phosphatase for microtubule-associated proteins [105]; focal adhesion kinase 1 which is a non-receptor protein-tyrosine kinase that plays an essential role in regulating cell migration and apoptosis [106,107]; protein tyrosine phosphatase type IVA 3 that stimulates progression from G1 into S phase during mitosis [108]; paxillin which is a cytoskeletal protein involved in actin-membrane attachment at sites of cell adhesion to the extracellular matrix [109]; proto-oncogene tyrosine-protein kinase Src that is a non-receptor protein tyrosine kinase [110]; signal transducer and activator of transcription 3 which mediates cellular responses to interleukins and other growth factors [111,112]; and signal transducer and activator of transcription 5A that is involved in signal transduction and activation of transcription [113].

## 3. Relationship between Post-Translational Modifications Associated with Colorectal Cancer

**Figure 2:**
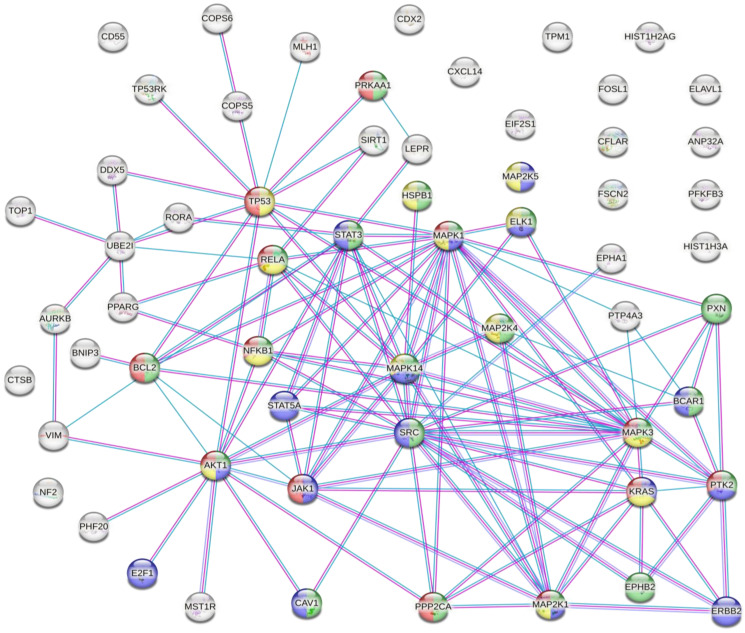
Protein–protein interaction network. Coloured nodes in green: proteins involved in the VEGFA-VEGFR2 signalling pathway. Coloured nodes in blue: proteins involved in the EGF-EFGR signalling pathway. Coloured nodes in red: proteins involved in the MAPK signalling pathway. Coloured nodes in yellow: proteins involved in the PI3K-Akt signalling pathway. Coloured nodes in grey: proteins that are not involved in any of the signalling pathways mentioned above. Edges represent protein–protein associations. Pink line: association experimentally determined. Blue line: association determined from curated databases. Purple line: protein homology.

The results of the analysis showed that there were several interactions between some of the proteins susceptible to inappropriate PTMs associated with CRC (Figure 2).

This analysis showed that there were strong interactions between TP53, AKT1, STAT3, STAT5A, JAK1, MAPK1, MAPK14, MAP2K1, and SRC. In fact, this network had significantly more interactions than expected, which means that proteins have more interactions among themselves than what would be expected from a random set of proteins, demonstrating that the proteins may be partially biologically connected as a group. This group of proteins is mainly involved in the PI3K-Akt, EGF-EFGR, MAPK, and VEGFA-VEGFR2 signalling pathways. On the one hand, PI3K-Akt is the classical signalling pathway involved in glucose metabolism that promotes cancer metabolic reprogramming by elevation of aerobic glycolysis (known as the “Warburg effect”) [114,115]. Both EGF-EGFR and MAPK signalling pathways are involved in proliferation, differentiation, and apoptosis. Its regulation in cancer cells allows the maintenance of proliferative signalling, promoting cancer cell survival [60,116]. On the other hand, VEGF and its receptors (such as VEGFR2) develop an important role in tumour-associated angiogenesis. This process is essential for tumour progression because it favours oxygen and nutrient uptake by cancer cells [117,118]. Therefore, the main PTMs identified in CRC are involved in cancer progression and cancer cell survival. The next step was to identify how the inadequate PTMs in these proteins associated with CRC may be modulated by plant-based dietary components.

## 4. Nutrigenomic Effects of Plant-Based Dietary Components on Protein Post-Translational Modifications Associated with Colorectal Cancer

The available scientific literature showed several plant-based dietary components that may modulate CRC-associated PTMs (Table 5). These components can be grouped according to bioactive compounds as follows: phenols, flavonoids, lignans, terpenoids/alkaloids, vitamins, phytochemicals, and plant extracts.

Among the different articles shown in Table 5, 39% of the studies evaluated PTMs in STAT3, 18% of the studies measured PTMs in AKT, 18% of the studies analysed PTMs in p53, 9% of the studies measured PTMs in ERK, and the rest of the studies analyzed PTMs in other proteins such as AMPK, BCL2, CDX2, EGFR, JAK2, EphA1, EphB2, PI3K, SCR, MLH1, and Nf-Kβ. All the studies referred to post-translational phosphorylation, and four of them also mentioned post-translational ubiquitination, acetylation, and methylation.

**Figure 3:**
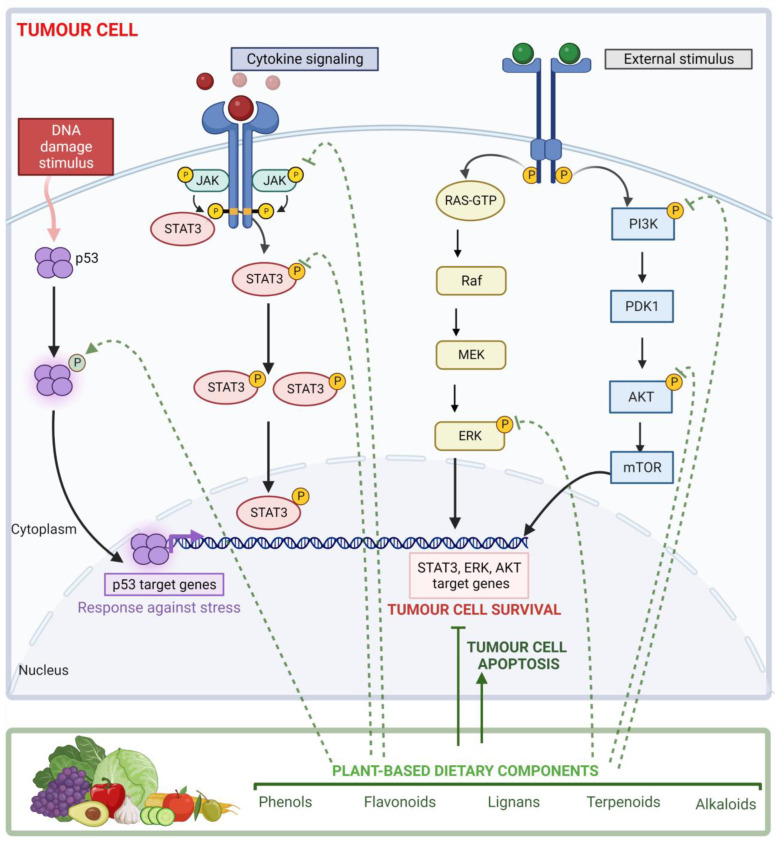
Effects of plant-based components on post-translational modifications associated with colorectal cancer. The first box lists the main signalling pathways that may be modulated through post-translational modifications by plant-based dietary components. From left to right, the p53, JAK/STAT, MAPK, and PI3K/AKT/mTOR signalling pathways are shown. The second box lists the main groups of bioactive compounds with modulatory activity on post-translational modifications. Arrow: promotes protein post-translational modification; no arrow: inhibits protein post-translational modification.

The PTMs induced by plant-based dietary components mainly consist of modulating those modifications observed in CRC (Figure 3). Concerning STAT3, tyrosine phosphorylation (Tyr 705) was upregulated in CRC. Several articles showed that some phenols, flavonoids, terpenoids, alkaloids, phytochemicals, and plant extracts were able to downregulate not only this phosphorylation but also STAT3 serine phosphorylation (Ser 727) in some cases [120,121,124,130]. Some of them are also involved in reduced phosphorylation of JAK2, which is upregulated in CRC [120,148,149,155,159]. Both proteins form the JAK/STAT signalling pathway, which has an important role in cytokine receptor signalling. In response to cytokines, its activation promotes immune cell division, survival, activation, and recruitment. This pathway not only participates in the immune response but also in the transcription of several genes involved in cell division and apoptosis regulation such as BCL2 [171,172].

In the case of AKT, serine phosphorylation (S473) was upregulated in CRC. Some studies revealed that harmine [151], ophiopogonin D [154], and luteolin [157] were able to downregulate this specific serine phosphorylation, while others such as coumarins [119], resveratrol [125], quercetin [140], secoisolariciresinol diglucoside [145], lycopene [158], and some plant extracts decrease protein phosphorylation [168,169]. Similarly, in PI3K and BCL2 proteins, quercetin, secoisolariciresinol diglucoside, and iodine-biofortified lettuce extract downregulated their phosphorylation, respectively [140,145,164]. On the other hand, delphinidin [136] and two different plant extracts [163,165] reverse carcinogen-induced phosphorylation of NF-kβ3 (Ser536). These proteins interact within the same pathway. The PI3K/AKT pathway participates in the modulation of cellular metabolism, cell growth, and apoptosis. PIK3K produces conformational changes and phosphorylation of AKT protein, inducing its activation. This cascade triggered the activation of NF-κB by enhancing the transcriptional activity of the p65 subunit, leading to apoptosis inhibition [173]. Likewise, the PI3K/AKT signalling pathway promotes the upregulation of Bcl-2 expression, which is considered an oncogene that inhibits apoptosis [174]. This suggests that plant-based dietary components may promote cancer cell apoptosis through downregulating AKT, PI3K, BCL2, and NF-kβ phosphorylation.

Another of the main proteins identified that can be modulated by plant-based dietary components is P53. This is considered a tumour suppressor involved in processes such as apoptosis, senescence, DNA repair, and cell cycle arrest [175]. The P53 pathway is activated against stress signals such as DNA damage. In response to this, P53 suffers from PTM and promotes the transcription of genes involved in cell response against stress [175]. These PTMs are mainly phosphorylation that is downregulated in CRC. However, several studies have shown that some plant-based dietary components such as phenols, flavonoids, terpenoids, and alkaloids could reverse it [123,126,128,135,137,139,140,146,147].

Concerning ERK1 and ERK2, both are part of a structurally related kinases family (MAPKs), whose signalling mechanism depends on an activating phosphorylation cascade. ERKs are central regulators of essential cellular functions such as cell differentiation, proliferation, migration, growth, survival, and metabolism [176]. Both proteins have post-translational phosphorylation upregulated in CRC, favouring cancer cell survival. As it has been shown in Table 5, some plant-based dietary components may downregulate their post-translational phosphorylation [130,156,160,161,166,169].

It is important to highlight that, due to the lack of human studies, the present review is focused on studies conducted in cells and murine models. Therefore, these findings cannot be transferable to other species. Future studies should address the connection between the modulation of PTMs associated with CRC elicited by plant-based dietary components in patients.

## 5. Conclusions

The available literature data suggest that different plant-based dietary components such as phenols, flavonoids, lignans, terpenoids, and alkaloids could prevent CRC development by targeting several molecular mechanisms such as the P53, JAK/STAT, PI3K/AKT, and ERK/MAPK pathways and affecting tumour behaviour through PTMs’ modulation in cell lines and murine models. The different signalling pathways affected during CRC development are mainly involved in cancer cell survival, and the main effects of plant-based dietary components are to promote apoptosis in tumour cells. Therefore, this could be a very interesting target for investigating the effect of supplementation based on these components as an adjuvant to chemotherapeutic, radiotherapeutic, and immunotherapeutic treatment in clinical trials. These findings will highlight the potential for precision nutrition strategies and the development of personalized nutritional plans in CRC treatment and may even serve as a basis for the development of dietary supplementation formulations for these patients to improve their prognosis and disease-free survival.

## Figures and Tables

**Table 3:** Post-translational phosphorylation associated with CRC.

Protein Name	Gene Name	Type	Ref
Acidic leucine-rich nuclear phosphoprotein 32 family member A	ANP32A	Upregulated	[61]
COP9 signalosome complex subunit 5	COPS5	Downregulated	[62]
Eukaryotic translation initiation factor 2 subunit 1	EIF2S1	Upregulated	[63]
Ephrin type-A receptor 1	EPHA1	Upregulated	[64]
Ephrin type-B receptor 2	EPHB2	Upregulated	[64]
Receptor tyrosine-protein kinase erbB-2	ERBB2	Upregulated	[65]
Heat shock protein beta-1	HSPB1	Upregulated	[66]
Tyrosine-protein kinase JAK1	JAK1	Upregulated	[67]
Mitogen-activated protein kinase 1	MAPK1	Upregulated	[69]
Mitogen-activated protein kinase 3	MAPK3	Upregulated	[68,69,70]
Mitogen-activated protein kinase 14	MAPK14	Upregulated	[71]
Dual specificity mitogen-activated protein kinase kinase 1	MAP2K1	Upregulated	[72]
Macrophage-stimulating protein receptor	MST1R	Upregulated	[73]
Merlin	NF2	Downregulated	[74]

**Table 5:** Effects of plant-based dietary components on PTMs associated with CRC.

Dietary Component	PTM	Target	Ref
Phenols			
Coumarins (8-methoxypsoralen)	↓ Phosphorylation	AKT1(Thr308)	[119]
Coumarins (fraxetin)	↓ Phosphorylation	STAT3 (Tyr 705); JAK2 (Tyr1007/1008)	[120]
Curcumin	↓ Phosphorylation	STAT3	[121]
Curcumin	↑ Ubiquitination	SIRT1	[122]
Resveratrol	↓ Phosphorylation	AKT1	[123]
Resveratrol	↓ Phosphorylation	STAT3 (Tyr 705, Ser727)	[124]
Resveratrol	↓ Phosphorylation	AKT1/AKT2	[125]
Polyphenols from lemon peel	↓ Phosphorylation	STAT3 (Ser 727)	[126]
Polyphenols from grape pomace	↑ Phosphorylation ↓ Methylation	P53 (Ser 20) CDX2 (5mC)	[127]
Polyphenols from grape seeds	↓ Phosphorylation	STAT3	[128]
Polyphenols from Annurca apple	↓ Methylation	MLH1	[129]
Olea europaea extract	↓ Phosphorylation ↓ Phosphorylation ↑ Phosphorylation	STAT3 ERK1 P53	[130]
Extra virgin olive oil	↓ Phosphorylation	STAT3 (Tyr 705)	[131]
Flavonoids			
Apigenin	↑ Phosphorylation	P53 (Ser15, Ser37)	[132]
Apigenin	↓ Phosphorylation	STAT3 (Tyr 705)	[133]
Baicalein	↓ Phosphorylation	STAT3 (Tyr 705)	[134]
Berry anthocyanidins	↓ Phosphorylation	SCR; EGFR	[135]
Blackberry anthocyanidins	↓ Phosphorylation	STAT3	[136]
Delphinidin	↓ Phosphorylation	STAT3 (Tyr 705)	[137]
Delphinidin	↓ Phosphorylation	NF-kβ3 (Ser536)	[138]
Luteolin	↑ Phosphorylation	P53 (Ser 15)	[139]
Quercetin	↓ Phosphorylation	PI3K; AKT	[140]
Sappanchalcone	↑ Phosphorylation	P53	[141]
Silibinin	↓ Phosphorylation	STAT3	[142]
Wogonin	↑ Phosphorylation ↑ Acetylation	P53 (Ser15) P53 (Lys380)	[143]
6,8-Diprenylorobol	↑ Phosphorylation	P53 (Ser15, Ser20, Ser46)	[144]
Lignans			
Secoisolariciresinol diglucoside	↓ Phosphorylation	PI3K; AKT1	[145]
Sesamin	↓ Phosphorylation	EphA1; EphB2	[64]
Terpenoids/Alkaloids			
Berberine	↓ Phosphorylation	STAT3	[146]
Berberine	↑ Phosphorylation	AMPK (Thr 172)	[147]
Berberine	↓ Phosphorylation	STAT3 (Tyr 705); JAK2	[148]
Carnosic acid	↓ Phosphorylation	STAT3 (Tyr 705); JAK2; SCR	[149]
Costunolide	↑ Phosphorylation	P53 (Ser15)	[150]
Evodiamine	↑ Phosphorylation	P53	[151]
Ginsenoside Rh2 (Ginseng)	↓ Phosphorylation	STAT3	[152]
Harmine	↓ Phosphorylation	AKT1 (Thr308, Ser473)	[153]
Ophiopogonin D	↓ Phosphorylation	AKT (S473)	[154]
Ursolic acid	↓ Phosphorylation	STAT3 (Tyr 705); JAK2 (Tyr1007/1008)	[155]
Vitamins			
Folic acid	↓ Phosphorylation	ERK1/2; SRC (Tyr416)	[156]
Phytochemicals			
Luteolin	↓ Phosphorylation	AKT1 (Ser473)	[157]
Lycopene	↓ Phosphorylation	AKT	[158]
Thymoquinone	↓ Phosphorylation	EGFR (Y1173); STAT3 (Tyr 705); JAK2	[159]
Plants extracts			
Cordyceps militaris	↓ Phosphorylation	ERK1/2 (Tyr 202/Tyr 204)	[160]
Dioscorea bulbifera	↓ Phosphorylation	ERK1/2 (Tyr 202/Tyr 204)	[161]
Foxtail millet (Setaria italica) cereal	↓ Phosphorylation	STAT3	[162]
Fresh tubers of Sagittaria trifolia L.	↓ Phosphorylation	NF-kβ3	[163]
Iodine-biofortified lettuce extracts	↓ Phosphorylation	BCL2	[164]
Mesua Assamica Kosterm extract	↓ Phosphorylation	STAT3; NF-kβ3	[165]
Nigella sativa	↓ Phosphorylation	ERK1	[166]
Paejangsan, Coix seed, and Mori Cortex	↓ Phosphorylation	STAT3 (Tyr 705)	[167]
Pharbitis semen seeds	↓ Phosphorylation	AKT	[168]
Trichosanthes kirilowii seeds	↓ Phosphorylation	AKT (Ser473); ERK1 (Thr202/Tyr204)	[169]
Withania somnifera	↓ Phosphorylation	STAT3	[170]

FER: feruloylacetone; 5mC: 5-methylcytosine. ↑: upregulation; ↓: downregulation.

## Data Availability

Not applicable.
